# Development of an Assessment Tool to Measure the Quality of Life Goal Setting for Cancer Survivors: A Content Validity Study

**DOI:** 10.7759/cureus.71272

**Published:** 2024-10-11

**Authors:** Katsuma Ikeuchi, Seiji Nishida, Mari Karikawa, Chiaki Sakamoto, Futoshi Mori, Mutsuhide Tanaka

**Affiliations:** 1 Department of Occupational Therapy, Faculty of Health and Welfare, Prefectural University of Hiroshima, Mihara, JPN; 2 Department of Nursing, Faculty of Health and Welfare, Prefectural University of Hiroshima, Mihara, JPN

**Keywords:** cancer rehabilitation, content validity, goal setting, quality of life, scale development, self-assessment tool

## Abstract

Introduction: An initial version of the Reengagement Life Goal Assessment Tool for Cancer Survivors (ReGAT-C) was designed to measure the quality of life goal-setting practice conducted by responsible healthcare professionals along with nonterminal cancer survivors undergoing inpatient cancer treatment. This study aimed to test content validity of the ReGAT-C and revise it.

Methods: Eleven experts and nine healthcare professionals participated in this study. Content validity assessments using questionnaires and focus group interviews were conducted with experts. Cognitive interviews were conducted with healthcare professionals. The content validity index was calculated based on expert questionnaires, and these interview data were analyzed by inductive and deductive approaches. When the ReGAT-C was substantially revised, it was retested through questionnaires.

Results: The initial version of the ReGAT-C was substantially revised and three new items were added, resulting in the development of a revised version of the ReGAT-C with 21 items. All participants re-evaluated the ReGAT-C, and the revised version was verified to have content validity.

Conclusions: The revised ReGAT-C would contribute to enabling healthcare professionals to assess whether they are based on collaboration with cancer survivors and multidisciplinary teams by reflecting on their own life goal-setting practice.

## Introduction

Cancer is known to incur various adverse physical, psychological, and social outcomes for many survivors [1,2]. Additionally, the cancer mortality rate has continuously decreased since 1991, resulting in an overall decrease of 33% [3], making it increasingly essential to enhance the quality of life (QoL) of cancer survivors.

Life goals give meaning to people’s lives, are an important part of identity development, and are defined as internal representations of the desired states that motivate behavior [4]. Life goals are often classified into categories, characteristics, and processes [4,5]. Life goal categories can be distinguished into health-related (e.g., physical health), psychological (e.g., inner psychological states), social (e.g., interpersonal relations), achievement-related (e.g., gaining social prestige), and leisure (e.g., intrinsically meaningful and self-rewarding activities) based on their nature [5]. Life goal characteristics refer to methods of describing goals, including goal content, life domains, importance, difficulty, attainability, intrinsic/extrinsic, and temporal ranges [6]. Life goal processes refer to the ways in which goals can be interacted with, such as the pursuit, loss, disruption, or adjustment of life goals [4].

As a life-threatening illness, cancer negatively influences one’s life goals, as well as physical, psychological, and social disabilities. For example, cancer survivors report fewer achievement-related and leisure goals and are known to often abandon difficult-to-attain or unattainable goals, and these issues can persist for as long as 18 months after diagnosis [5]. Conversely, when cancer survivors experience more progress in achieving goals or downgrade the importance of unattainable goals, they are less likely to experience reductions in their global QoL over time [7]. These findings indicate that healthcare professionals should provide appropriate support to cancer survivors on how to deal with their life goals.

The term goal setting refers to a formal process in which a rehabilitation professional or team negotiates goals together with the patient and/or their family [8] and is considered a core component of cancer rehabilitation interventions [9]. A popular tool used for goal setting is goal attainment scaling (GAS) [10,11], which serves to develop personalized rating scales for each patient. The general evaluation procedure is as follows: (1) agreement with the patient on important goals; (2) expressing goals and developing milestones; (3) weighting goals based on importance and/or difficulty; (4) evaluating outcomes. The GAS measures goal attainment individually, can be used as an outcome measure, and enhances the application of a person-centered approach in the rehabilitation process [12]. Despite these advantages, the GAS also has the following challenges: patients often get confused about the process and feel uncomfortable talking about negative outcomes [13]; the goal attainment of cancer survivors as measured by GAS is not associated with QoL [14]; the GAS calculates goal attainment in the aspects of partial aspects of life goals, such as goal importance and/or difficulty; it was not developed for cancer survivors.

To our knowledge, there is a lack of practical reports and research on goal-setting tools for cancer survivors compared to other diseases, such as acquired brain injury. This may be due to the aforementioned challenges of the GAS. We have hence designed the Reengagement Life Goal Assessment Tool for Cancer Survivors (ReGAT-C), an easy-to-apply assessment tool that can evaluate the life goals of cancer survivors based on multiple aspects. However, the validity and reliability of the ReGAT-C have not yet been verified. Hence, this study aimed to check the ReGAT-C for content validity and revise it.

## Materials and methods

Concept development and item generation

The ReGAT-C development group consisted of occupational therapists (OTs; first, second, and fourth authors), a registered nurse (RN; third author), and a neurosurgeon (fifth author). The components of the ReGAT-C relate to the quality of life goals-setting practice based on the collaboration between three types of healthcare professionals, namely physical therapists (PTs), OTs, and RNs along with nonterminal cancer survivors undergoing inpatient cancer treatment and with the expectation of hospital discharge. Compared to cancer survivors in the early stages of the disease, late-stage cancer survivors (i.e., within one year prior to death) have been shown to change goals more often [15]; therefore, the ReGAT-C targets only nonterminal cancer survivors. In Japan, terminal patients are generally considered to be individuals whose prognosis for life is expected to be less than six months. In this study, we defined nonterminal cancer survivors as individuals whose prognosis for life is expected to be six months or more.

We conducted two literature reviews to generate the candidate item pool for the ReGAT-C, as this process should be done prior to content validity tests. The first literature review involved a comprehensive search for papers reporting on rehabilitation practices, including life goal setting for cancer survivors, and described the elements related to goal domains, characteristics, and processes, which are the life goal practice in the 24 included papers [16]. The second literature review explored the factors influencing individual performance after goal setting by reviewing papers on rehabilitation practices that applied the goal-setting theory proposed by Lock and Latham in 1990 [17]. The main findings of this review were the various factors that can influence survivors’ performance after goal setting and the choice of setting learning and performance goals according to the survivors’ clinical situation. A learning goal is a goal related to the desired number of strategies, processes, or procedures to be developed to master a task, such as “discover and implement how to increase the number of steps,” and a performance goal is a goal to the achievement of specific tasks according to certain standards of proficiency, such as “perform aerobic exercise for more than 150 minutes.”

Based on the findings of these literature reviews and in-depth discussions in the development group, the ReGAT-C was developed, comprising 18 items corresponding to the three subscales of classification (three items), characteristic (nine items), and process (six items; Table 1). After healthcare professionals set life goals with a cancer survivor, professionals (i.e., respondents) reflected on the practice and self-assessed these items on a five-point scale for a maximum of three life goals while referring to the developed ReGAT-C manual. During the performance of the tool, if the respondent had set multiple life goals and some of these goals applied to items to a different degree, the response option must be selected according to the goal with the highest degree of applicability. Because the ReGAT-C has items on the cancer survivors' experience (e.g., items 4 and 5), the respondents had to adequately recall their life goal-setting practices before answering the question.

**Table 1:** Initial version of the Reengagement Life Goal Assessment Tool for Cancer Survivors (ReGAT-C) ^a^ Classification A: health-related, psychological, social, achievement-related, and leisure goals. ^b^ Classification B: learning goals and performance goals. ^c^ Conditions that should be met for Classification B: learning goals should be set rather than performance goals when at least one of the following three conditions is met: (i) low cognitive functioning and motivation in the cancer survivor; (ii) the difficulty of the tasks is so high that failure to achieve the life goals is likely to be viewed as a major failure by the cancer survivor and may lead to pessimism; (iii) when the cancer survivor is not yet active, such as at the start of rehabilitation.

Subscales	Items	Response options
Classification	1. Number of Classification A^a^ include in the life goals.	1: 1 unit, 2: 2 units, 3: 3 units, 4: 4 units, 5: 5 units
	2. The life goals you set can be clearly distinguished when checking the contents of Classification B^b^.	1: Strongly disagree, 2: Disagree a little, 3: Neither agree nor disagree, 4: Agree a little, 5: Strongly agree
	3. You can set life goals considering the conditions that should be met for goal setting outlined in Classification B^c^ in the manual.	
Characteristic	4. There are life goals that the cancer survivor wants to engage in.	
	5. There are life goals that the cancer survivor enjoys engaging in.	
	6. There are life goals to objectively assess if improvement is possible or not.	
	7. There are life goals that specifically define when, who, and how.	
	8. There are life goals for which people around the cancer survivor are glad when she/he engage in them.	
	9. There are life goals that you think are achievable.	
	10. There are life goals that the cancer survivor thinks are achievable.	
	11. Low risk of pathological fractures in case of bone metastasis	
	12. There are life goals that multidisciplinary professionals find valid for their content.	
Process	13. After life goals were established, they were reassessed for necessary modifications or additions.	1: Not reassessed at all, 2: Slightly reassessed, 3: Reassessed once, 4: Reassessed twice, 5: Reassessed three or more times
	14. You can explain to others why you have set these life goals.	1: Strongly disagree, 2: Disagree a little, 3: Neither agree nor disagree, 4: Agree a little, 5: Strongly agree
	15. The cancer survivor can realize improvements in their abilities by engaging in their life goals.	
	16. You write down life goals on paper, computer, or smartphone with the cancer survivor.	
	17. You reflect on the course and progress of life goals with the cancer survivor.	
	18. The cancer survivor can recall what their life goals were.	

Design and procedures

**Figure 1:**
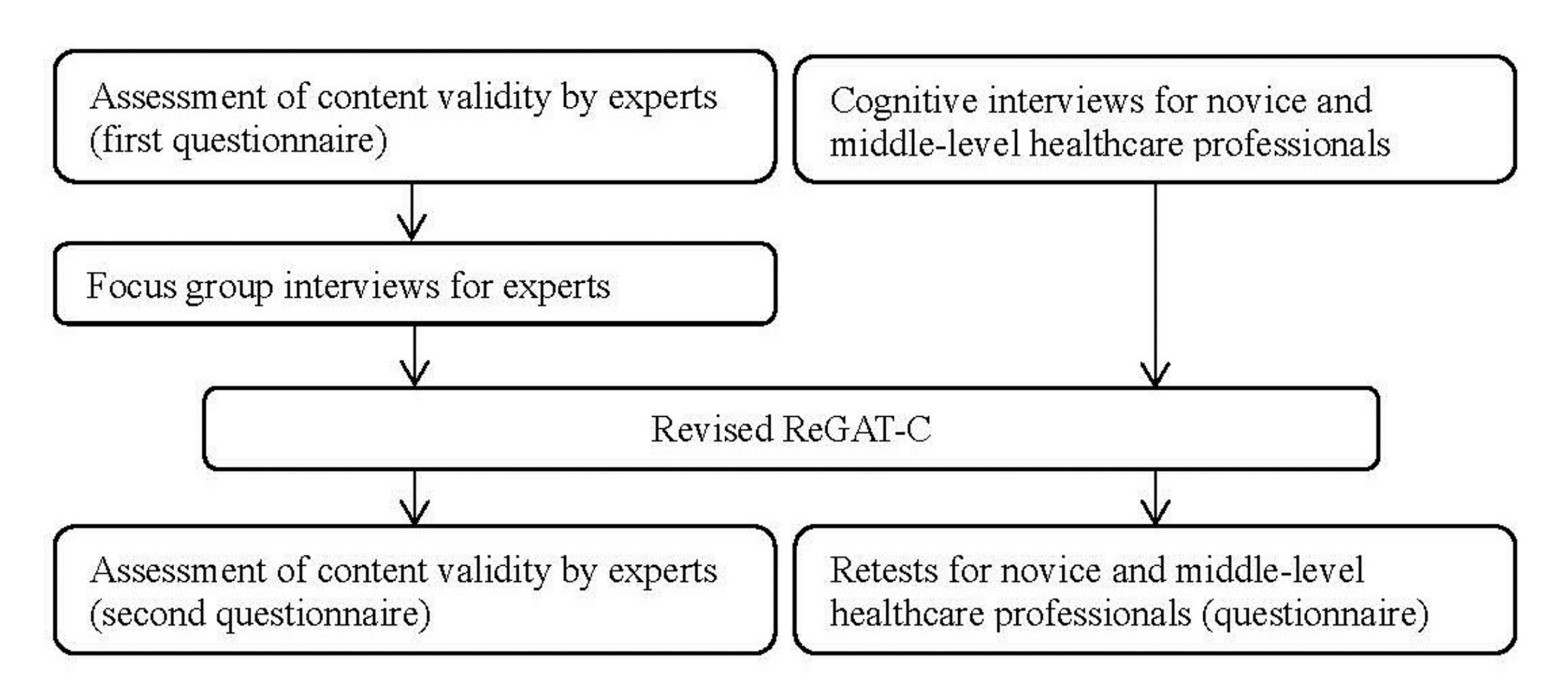
Procedures conducted for content validity testing ReGAT-C: Reengagement Life Goal Assessment Tool for Cancer Survivors

The study was conducted using quantitative and qualitative data through the following procedures (Figure 1).

The procedures outlined in Figure 1 were designed based on the user manual of the Consensus-based Standards for the Selection of Health Measurement Instruments (COSMIN) methodology for assessing the content validity of outcome measures [18], and the Standards for Reporting Qualitative Research (SRQR) [19], which aims to improve qualitative research transparency in all of its aspects. The COSMIN lists relevance (i.e., all items should be relevant for the construct of interest within a specific population and context of use), comprehensiveness (i.e., no key aspects of the construct should be missing), and comprehensibility (i.e., items should be understood by users as intended) as points to be considered when validating content validity. Furthermore, the COSMIN outlines that data collection on relevance and comprehensiveness should be extracted from experts and respondents (PTs, OTs, and RNs in this study), while comprehensibility requires data from respondents [18]. Therefore, this study collected quantitative data from experts to objectively assess content validity. Qualitative data were also collected to revise the ReGAT-C based on the opinions of experts and respondents because the ReGAT-C was designed solely on the basis of consensus within the development group.

Data collection and analysis

Assessment of Content Validity by Experts (First Questionnaire)

PT, OT, and RN experts were recruited through purposive sampling. We recruited at least three experts from each profession because COSMIN recommends that more than seven experts participate [18]. The inclusion criteria for experts were as follows: (1) have set life goals in collaboration with cancer survivors; (2) have clinical practice for at least one cancer survivor per month; (3) be qualified as certificated PT, OT, or RN by the relevant Japanese professional association; and (4) have clinical experience with cancer survivors at an inpatient institution for at least 10 years. The recruitment of experts was conducted with care to avoid bias regarding the age of the cancer survivors which they usually cared for and the cancer types with which they had experience.

Experts who agreed to participate in the study were mailed the ReGAT-C manual, a form to complete it, and a form to complete the content validity assessment. The experts rated each of the 18 items comprising the ReGAT-C on a four-point scale (1 = not relevant, 2 = somewhat relevant, 3 = quite relevant, 4 = highly relevant) according to two questions (question A: is this item relevant for the components in this study?, and question B: is this item relevant for the target population of cancer survivors in this study?), and returned the assessment forms.

The item-level content validity index (I-CVI), scale-level content validity index, and averaging calculation method (S-CVI/ave) were calculated for the expert ratings [20,21]. The I-CVI was calculated as the number of experts who answered “quite relevant” or “highly relevant” divided by the total number of experts, and the S-CVI/ave was the average value of I-CVI. The I-CVI and S-CVI/ave were calculated independently for questions A and B, implying that 36 I-CVI and 2 S-CVI/ave were calculated for the 18 items. The I-CVI and S-CVI/ave standards were defined as 0.78 or higher and 0.90 or higher [20,21], respectively; if the values fell below these standards, we considered that the corresponding ReGAT-C manual and/or items had to be revised, and focus group interviews (FGI) were applied to tackle the issue through making use of qualitative data.

FGI for Experts

After the experts answered the first questionnaire, FGIs were conducted using the Zoom web conferencing system (Zoom Video Communications, Inc., San Jose, USA). There would initially be three FGIs, once for each professional group of PTs, OTs, and RNs. This is because participant homogeneity is required to elicit group dynamics [22]. However, two FGIs were conducted for the OTs, dividing them into two groups of two each, as there were no dates available for all four OTs to participate at one time. Therefore, there were four FGIs in total in the study. The interviewers for the FGI with OTs and PTs were the first author (i.e., the ReGAT-C development manager of an OT); for the FGI with RNs, the interviewers were the first author and the third author (i.e., an RN). Both the first and third authors had multiple experiences with qualitative studies. Half of the expert participants had interacted with the interviewer (first author) several times before the FGI.

The FGI guide (Table 2) included a question asking why the expert answered “not relevant” or “somewhat relevant” in the first questionnaire. Furthermore, questions regarding the comprehensiveness, notations, and understanding of the ReGAT-C were included. To elicit group dynamics among experts, the screen of the interviewer’s PC was shared such that all experts and interviewers could view the progression chart, the interviewer’s questions, and a brief summary of the experts’ statements [23]. Whenever one of the experts presented an opinion in the FGI, the interviewer asked the other participating experts if there was anyone who had similar or different experiences regarding a specific opinion; this served to avoid the interviewer directly responding to the experts’ opinions, as this could potentially inhibit responses or evoke biases [24]. The FGIs were recorded using Zoom’s recording function and later converted into verbatim transcripts.

**Table 2:** Focus group interview guide for experts ReGAT-C: Reengagement Life Goal Assessment Tool for Cancer Survivors, Question A: Is this item relevant for the components in this study?, Question B: Is this item relevant for the target population of cancer survivors in this study?

Categories	Question
Relevance for the components	You (an expert) answered “not relevant” or “somewhat relevant” for item X on the ReGAT-C in response to Question A. Please tell us the reason for this.
Relevance for cancer survivors	You (an expert) answered “not relevant” or “somewhat relevant” for item X on the ReGAT-C in response to Question B. Please tell us the reason for this.
Comprehensiveness	Do you think that the items included in the ReGAT-C cover the full range of components set in this study? If there are any aspects that are lacking, please tell us.
Notations and understanding	What did you think of the wording, response options, readability of the layout, font size, and length of the ReGAT-C? If you find anything that is difficult to understand or needs to be corrected, please tell us.

The data was analyzed by distinguishing between each item of ReGAT-C and using a combination of inductive and deductive approaches. An inductive approach was applied to the coding process. A deductive approach was applied while naming categories after creating them based on the similarity of the codes. Since the categories of “relevance for the components,” “relevance for cancer survivors,” “comprehensiveness,” and “notations and understanding” were identified in advance for the FGI guide, these categories were applied deductively while naming the categories.

Furthermore, the process was carefully followed in a reflexive manner by repeatedly recreating the code or category and re-extracting the data. The analysis was conducted based on a consensus among the first (an OT, M.S. in Health and Welfare Science), second (an OT, PhD in Health Science), and third authors (an RN, PhD in Nursing), all of which were involved in the analyses and had experience with multiple qualitative studies.

Cognitive Interviews for Novice and Middle-Level Healthcare Professionals

Novice and middle-level healthcare professionals (PTs, OT, and RNs) were recruited through purposive sampling. We recruited at least three practitioner respondents from each profession to participate, as COSMIN recommends more than seven. The inclusion criteria were as follows: (1) have set life goals in collaboration with cancer survivors; (2) have clinical practice with at least one cancer survivor per month; (3) not qualified as a certified PT, OT, or RN by the relevant Japanese professional association. Practitioner respondents were recruited carefully to avoid significant bias in the number of years of experience working with cancer survivors, the number of cancer survivors for which they had set life goals, the age of the cancer survivors they usually cared for, and the cancer types they had experience with.

Practitioner respondents who agreed to participate in the study were invited to participate in individual face-to-face cognitive interviews. A cognitive interview is a general method for evaluating data collection instruments (e.g., questionnaires, brochures, and cover letters), and can be used to investigate how target audiences understand, mentally process, and respond to materials, with a special emphasis on potential breakdowns in this process [25]. Namely, the cognitive interviews were used for collecting data on comprehensibility.

The first author worked as the interviewer after careful planning because no researcher in the ReGAT-C development group had experience with cognitive interviews. After a preliminary practice session that served to enable practitioner respondents to voice more detailed information, they were asked to voice their thoughts and understanding as they read each item of the ReGAT-C using the think-aloud technique while filling out the form.

Three techniques were used in the interviews, namely think-aloud, observation, and retrospective probing. The procedures involved a cognitive interview immediately after the practitioner respondents completed the ReGAT-C. Practitioner respondents completed each item on the ReGAT-C while using the think-aloud technique. The interviewer observed them completing the ReGAT-C, and then conducted a cognitive interview using an interview guide that included a retrospective probing procedure corresponding to the four stages from Tourangeau’s cognitive model, as follows: comprehension of the question - comprehend what is being asked; retrieval from memory of relevant information - retrieve prior knowledge from memory; decision processes - make a judgment to find the answer; and response processes - either select a pre-defined category that fits the answer or find appropriate words to express the response [26]. The interviewer’s questions were administered according to a cognitive interview guide (Table 3), which encompasses questions on comprehensibility, relevance for the components, relevance for cancer survivors, comprehensiveness, and notations. The cognitive interviews were recorded using an IC recorder and later converted into verbatim transcripts.

**Table 3:** Cognitive interview guide used for novice and middle-level healthcare professionals ReGAT-C: Reengagement Life Goal Assessment Tool for Cancer Survivors; FGI: focus group interviews

Categories	Procedure
Comprehensibility	Identifying items that practitioner respondents had difficulty understanding or for which the response provided went against the ReGAT-C developers’ expectations through the following procedures: Asking about when practitioner respondents found it difficult to answer items. Observation of the response (e.g., whether the practitioner respondents took time to answer, hesitated, sighed, or scratched own head when answering). Think-aloud technique. Asking practitioner respondents why they had difficulty understanding or how they understood items, and what situations they recalled while answering the question. Identifying the stage, based on Tourangeau’s four stages (comprehension of the question, retrieval from memory of relevant information, decision processes, and response processes), where the error is thought to have occurred, and asking the practitioner respondent questions using the probes corresponding to that stage. The probes corresponding to Tourangeau’s four stages are as follows: Comprehension of the question: what does this question mean to you? Please explain in your own words. Retrieval from memory of relevant information: how well did you recall your practice when answering this question? Decision processes: how certain do you think you are of your answer to this question? Why do you think so? Response processes: how did you choose your answer to this question?
Relevance for the components	Same as the FGI guide (Table 2) conducted for experts.
Relevance for cancer survivors	
Comprehensiveness	
Notations	

An inductive and deductive approach similar to that used for the expert FGI was used for the data analysis of cognitive interviews. Since the categories of “comprehensibility,” “relevance for the components,” “relevance for cancer survivors,” “comprehensiveness,” and “notations” were identified in advance for the cognitive interview guide, these categories were applied deductively while naming the categories. All the above qualitative analyses were executed using NVivo 14, a qualitative data analysis software (QSR International, Melbourne, Australia).

Revised ReGAT-C

Regarding the items for which the I-CVI was below the standard, whether the items should be revised or deleted was discussed based on the results of the qualitative analysis obtained from the expert FGIs and cognitive interviews. Even if the items had an I-CVI above the standard, whether they should be revised was discussed if the results of the qualitative analyses indicated the need for revision. The tool’s response options and manual were revised in parallel to the revision of the items, yielding a revised version of the ReGAT-C. All revisions were made after all authors reached consensus on them.

Retests for Novice and Middle-Level Healthcare Professionals (Questionnaire)

The revised ReGAT-C version, a document describing the contents of and the reasons for the revisions, and a questionnaire were mailed to the practitioner respondents who participated in the cognitive interview. The questionnaire included the following questions: whether the revised version of the ReGAT-C reflects what the practitioner respondent pointed out during the cognitive interview; whether all the revised parts of the ReGAT-C are understandable to the practitioner respondent; whether the practitioner respondent agrees with the revised content of the ReGAT-C, considering the experiences of the practitioner respondent and people around them.

Assessment of Content Validity by Experts (Second Questionnaire)

The same experts who participated in the first content validity procedure and FGIs were mailed the revised ReGAT-C version, a document with the contents of and the reasons for the revisions, and a questionnaire to reassess content validity. They reassessed the relevance of the components and the relevance for cancer survivors on the same four-point scale used in the first questionnaire. The I-CVI and S-CVI/ave values were calculated based on the results.

Ethical considerations

This study was approved by the Research Ethics Committee of the Prefectural University of Hiroshima (approval number: issue 23MH014). Prior to the questionnaires and interviews, we explained to all experts and novice and middle-level healthcare professionals, that they could refuse to participate in the study or withdraw during the course of the study and then obtained their written consent.

## Results

Assessment of content validity by experts (first questionnaire)

Eleven experts (four PTs, four OTs, and three RNs) participated in the process (Table 4). The years of experience working with cancer survivors ranged from 11 to 21 years. Most experts (n = 9 or more) routinely cared for cancer survivors over the age of 40 years. All the experts had clinical experience with various cancer types. Five experts had set life goals for 50-99 cancer survivors, and five had set life goals for more than 100 cancer survivors.

**Table 4:** Characteristics of experts OT: occupational therapist; PT: physical therapist; RN: registered nurse

Characteristics	OT (n = 4)	PT (n = 4)	RN (n = 3)
Years of experience working with cancer survivors Median (range)	17 (13-21)	13 (11-19)	14 (11-19)
Age of the cancer survivors that they usually cared for (n)			
18-39	0	3	0
40-64	2	4	3
65-74	4	4	3
75+	3	4	3
Number of cancer survivors for which they set life goals (n)			
25-49	1	0	0
50-74	1	2	1
75-99	1	0	0
100+	1	2	2
Cancer types experienced (n)			
Head (brain tumor)	4	3	3
Head and Neck	2	2	3
Lung	3	4	3
Breast	4	3	3
Gynecology	3	3	3
Pancreas	3	4	3
Gallbladder	4	3	3
Liver	4	4	3
Digestive	4	4	3
Skin	2	0	3
Urological	4	3	3
Hematopoietic	4	4	3
Sarcoma	2	2	2

Experts evaluated the ReGAT-C’s 18 items, resulting in an I-CVI range of 0.64-1.00, with four items below the standard value (Table 5). Specifically, item 2’s relevance for the components (0.73) and relevance for cancer survivors (0.73), item 3’s relevance for the components (0.64), item 15’s relevance for cancer survivors (0.73), and item 16’s relevance for cancer survivors (0.73) were below the I-CVI standard. The S-CVI/ave was 0.88 and 0.87 for relevance for the components and relevance for cancer survivors, respectively; these figures were slightly below the standard.

**Table 5:** Item-level content validity index The following two questions were asked to experts, question A: is this item relevant for the components in this study?” (relevance for the components); question B: “is this item relevant for the target population of cancer survivors in this study?” (relevance for cancer survivors). Items that are below the standard are shown with an asterisk (*). I-CVI: item-level content validity index

Item	I-CVI (first)	I-CVI (second)
1-A	0.91	1.00
1-B	1.00	1.00
2-A	0.73*	1.00
2-B	0.73*	0.91
3-A	0.64*	1.00
3-B	0.91	0.91
4-A	0.91	1.00
4-B	1.00	1.00
5-A	0.82	1.00
5-B	0.91	1.00
6-A	0.91	1.00
6-B	0.82	1.00
7-A	0.91	1.00
7-B	0.82	1.00
8-A	0.82	1.00
8-B	0.82	1.00
9-A	1.00	1.00
9-B	0.91	1.00
10-A	1.00	1.00
10-B	1.00	1.00
11-A	0.91	1.00
11-B	0.91	1.00
12-A	0.82	1.00
12-B	0.82	1.00
13-A	1.00	1.00
13-B	0.91	1.00
14-A	1.00	0.91
14-B	0.91	0.91
15-A	0.82	0.91
15-B	0.73*	1.00
16-A	0.82	1.00
16-B	0.73*	1.00
17-A	0.91	1.00
17-B	0.91	1.00
18-A	1.00	1.00
18-B	0.82	1.00
19-A	None	1.00
19-B	None	1.00
20-A	None	1.00
20-B	None	1.00
21-A	None	0.91
21-B	None	0.91

FGI for experts

The FGIs took 56 min for PTs, 52 and 43 min for OTs, and 73 min for RNs. The qualitative analysis results were categorized into four categories.

Relevance for the Components

The experts commented on three of the 18 items in the ReGAT-C regarding their relevance for the components. Regarding item 2, there were comments that simply distinguishing between learning and performance goals does not lead to life goal-setting practice improvements.

Regarding item 8, there were remarks that it is not necessarily important for people around cancer survivors (e.g., family members) to be “glad,” and that it is instead more important that they “agree” with the contents of the set life goals.

Regarding item 11, there were descriptions that it would be desirable to make it an item that allows the examination of the relationship between behavior and risk, since users may respond only on the basis of whether they have bone metastasis, rather than on the behavior of cancer survivors toward their life goals. Furthermore, experts commented that the question was difficult to answer and that even an orthopedic surgeon could find it challenging to establish the degree of pathological fracture because it is often unclear.

Relevance for Cancer Survivors

Experts talked about five of the 18 items in the ReGAT-C regarding relevance for cancer survivors.

Regarding item 4, there were comments that even if the user directly asks the cancer survivor if one wants to engage in these life goals, the cancer survivor may find it difficult to answer their own true feelings.

Regarding item 5, there were comments that some cancer survivors may have feelings of “fight” rather than “enjoy,” and that such cancer survivors often enjoy the process of engaging in life goals rather than engaging in them.

Regarding item 10, there were comments that this item could not be rated correctly for cancer survivors who had prominent defense mechanisms in place, such as denial.

Regarding item 13, there were comments that if the period of hospitalization is short because the treatment is smooth, or if the Eastern Cooperative Oncology Group (ECOG) Performance Status (PS) scale remains unchanged at a high level throughout the cancer treatment period, there is little need to review life goals. Namely, they believed that a similar option to items 14-18 in the initial version of the ReGAT-C would be preferred because the frequency of life goal review is likely to vary by cancer survivor.

Regarding item 15, there were comments that there are scenarios where the cancer survivors could not realize improvements in their abilities; for example, in cases where the cancer survivor’s skill is already too high, or where there is no expectation of the abilities recovering to the same level as that before treatment. Furthermore, there were comments that a “feeling of accomplishment or satisfaction” was more important than “realization of ability improvements” to improve the QoL of cancer survivors, as it is not always necessary for patients to experience ability improvements for their QoL to enhance.

Comprehensiveness

The experts proposed two points regarding comprehensiveness. First, it is important to set life goals that are compatible with cancer treatment after discharge because the ReGAT-C targets cancer survivors who participate in rehabilitation with the aim of being discharged from the hospital. Second, it would be valuable to include an item to confirm the degree of discussion between cancer survivors and healthcare professionals as it varies by case. Third, they suggested that it would be easier to answer the question if it was described with terms related to “positive” rather than “enjoyment.”

Notations and Understanding

Experts remarked the following regarding the notations and understanding. Regarding item 3, there were comments that it is difficult to imagine the conditions under which it is recommended to set learning goals rather than performance goals.

Regarding item 6, it was seemingly unclear what kind of improvement is indicated by “improvement is possible or not.”

Regarding item 12, there were descriptions that the desirable number and scope of multidisciplinary professionals were unclear; in particular, the word “multidisciplinary” would be interpreted as a description that multiple professionals should consider the life goals procedures to be valid. There were also comments that it is more important that at least one professional of a different type other than a user agrees with the content of the goal than that multidisciplinary professionals find valid for the content of the goals.

Regarding item 15, there were discussions about a lack of clarity as to what kind of improvement is indicated by “improvement in their abilities.”

Regarding item 16, there were descriptions that a specific explanation was needed concerning the act of writing down life goals, such as whether it was the cancer survivor or the healthcare professional who wrote them down.

Regarding all items of the tool, there were comments that it would be easier to answer them if the items with the same subject were placed closer to each other, as each item contained a subject for both cancer survivors and healthcare professionals.

Cognitive interviews for novice and middle-level healthcare professionals

Nine novice and middle-level healthcare professionals of practitioner respondents (three PTs, three OTs, and three RNs) agreed to participate in this study. Their characteristics are shown in Table 6. Their years of experience working with cancer survivors ranged from 1 to 17 years, and most (n = 7) had routinely worked with cancer survivors aged over 65 years. The number of cancer survivors for whom life goals were set ranged from less than 25 (n = 5) to more than 100 (n = 2).

**Table 6:** Characteristics of novice and middle-level healthcare professionals OT: occupational therapist; PT: physical therapist; RN: registered nurse

Characteristics	OT (n = 3)	PT (n = 3)	RN (n = 3)
Years of experience working with cancer survivors: Median (range)	5 (1-11)	8 (2-11)	10 (6-17)
Age of cancer survivors that they usually cared for (n)			
18-39	1	1	1
40-64	1	1	1
65-74	3	2	2
75+	2	3	3
Number of cancer survivors for which they set life goals (n)			
1-24	1	1	3
25-49	0	1	0
50-74	1	0	0
75-99	0	0	0
100+	1	1	0
Cancer types experienced (n)			
Head (brain tumor)	3	3	0
Head and neck	1	1	0
Lung	2	2	1
Breast	2	2	2
Gynecology	2	2	0
Pancreas	2	2	2
Gallbladder	2	2	1
Liver	2	2	2
Digestive	2	2	3
Skin	1	1	0
Urological	2	1	1
Hematopoietic	2	2	0
Sarcoma	2	1	1

The characteristics of cancer survivors for which practitioner respondents filled out the ReGAT-C are shown in Table 7. The average time that it took for them to complete the ReGAT-C was 15 minutes 37 seconds (range: 6 minutes 20 seconds to 25 minutes 30 seconds). Most cancer survivors were female (n = 6), over 65 years of age (n = 8), stages III-IV (n = 6), and had undergone surgery (n = 7) or chemotherapy (n = 5). They had varying the ECOG PS scale.

**Table 7:** Characteristics of cancer survivors as reported on ReGAT-C by novice and middle-level healthcare professionals ^a^ The total is not nine because there were survivors with multiple cancer types. ReGAT-C: Reengagement Life Goal Assessment Tool for Cancer Survivors; ECOG PS: Eastern Cooperative Oncology Group Performance Status

Characteristics	n = 9
Sex	
Male	3
Female	6
Age	
18-39	0
40-64	1
65-74	6
75+	2
Cancer types^a^	
Head (brain tumor)	2
Breast	1
Gallbladder	1
Digestive	3
Hematopoietic	2
Sarcoma	3
Stage	
I	1
II	2
III	3
IV	3
Cancer treatment	
Surgery	7
Chemotherapy	5
Radiotherapy	3
ECOG PS scale	
0	1
1	3
2	1
3	2
4	2

The results of the qualitative analysis were categorized into the following five categories.

Comprehensibility

Practitioner respondents commented on topics about 12 of the 18 items in the ReGAT-C regarding comprehensibility. Regarding item 1, there were comments about confusion as to whether the number of Classification A entries to be considered should reflect the time of setting the life goals or the time of answering the ReGAT-C, i.e., after a certain period had elapsed since the life goals were set.

Regarding items 2 and 3, there were comments that the practitioner respondents had no experience distinguishing between learning and performance goals, and therefore took time to answer these questions. Furthermore, the practitioner respondents were confused as to whether the distinction outlined in Classification B and the consideration of the conditions for applying Classification B should be made while considering the life goals set at the time of setting the life goals or at the time of answering the ReGAT-C (i.e., after a certain period had passed since setting the life goals).

Regarding item 5, there were descriptions of a lack of clarity as to whether feelings similar to enjoyment (e.g., positivity, joy, and satisfaction) experienced by cancer survivors should be included in the enjoyment category.

Regarding item 6, there were discussions that it was unclear whether the person making the objective evaluation was to be a healthcare professional or a cancer survivor. Furthermore, they commented that healthcare professionals get in touch with cancer survivors during hospitalization, making it impossible for them to know if some life goals can be improved after discharge.

Regarding item 10, practitioner respondents were unsure about how to answer the item when cancer survivors had not been asked whether it was achievable because of patient anxiety or demotivation.

Regarding item 12, there were comments that the term “multidisciplinary” made it difficult to understand how many specific professionals or types of professionals needed to be considered for the life goal practice to be valid.

Regarding item 13, practitioner respondents were confused about the extent to which modifications/additions to life goals should be counted as actual modifications/additions. There was also a comment that they were unsure as to how to answer the item when the life goal had not been modified, but the intervention had been modified.

Regarding item 14, the practitioner respondents were unsure whether the explanation should be given to physicians or to the cancer survivor and one’s key persons, which made it difficult for them to answer the item.

Regarding item 15, there were comments that when life goals which is a feasible activity after discharge (e.g., traveling) were set, practitioner respondents were unsure of what to answer because the cancer survivors often did not realize any improvement in their abilities during hospitalization.

Regarding item 16, there were comments that it was difficult to understand what kind of situation should be imagined to answer the item. In particular, it was considered unclear whether the written life goals should be visible to the cancer survivor or whether they should be adequately shared with the cancer survivor, as the term “with” is included in the item.

Regarding item 17, there were comments that they were unsure as to how to answer this item because the goal they set with the cancer survivor was “travel,” which is an activity to be performed after discharge, implying that they would not be able to reflect on the course and progress of the life goal with the survivor.

Relevance for the Components

Practitioner respondents provided comments about three of the 18 items in the ReGAT-C regarding their relevance for the components. Regarding item 12, it was noted that the practitioner respondents felt strange about the lower ReGAT-C scores assigned to this item when cancer survivors had life goals that were relatively easy to achieve because users had less need to share their goals with multidisciplinary professionals.

Regarding item 13, there were comments that practitioner respondents felt it strange that their ReGAT-C scores would be reduced because of reviewing life goals only once when they deemed that this single review was sufficient for the cancer survivor they have worked with.

Regarding item 16, there were discussions that although it is important to write down goals such that they can be shared between cancer survivors and healthcare professionals, it is unlikely that writing down life goals on paper or smartphones would affect life goal-setting quality.

Relevance for Cancer Survivors

No practitioner respondents talked about the relevance for cancer survivors.

Comprehensiveness

Practitioner respondents discussed two points regarding comprehensiveness. First, regarding the quality of the process of setting life goals, it was suggested that a new item be created to ascertain whether the cancer survivor is satisfied with the achievement of the goal. Second, it was proposed that a new item on collaboration with family members be added to ascertain whether life goals include the family’s expectations, whether the family knows about the goals, and whether the family is cooperating toward or engaging in goal achievement.

Notations

Two topics regarding notations were identified. First, practitioner respondents commented that item 11 on bone metastasis was not clearly related to item 10; thus, it took more time for them to understand this item. They proposed revising the item order. Second, there was no consistency across practitioner respondents regarding compliance with the rule that when respondents set multiple life goals and the degree to which each goal applied to an item differed, they chose the option related to the goal with a higher degree of applicability.

Revised ReGAT-C

Based on the above comments, the authors thoroughly discussed the revision of the ReGAT-C, which unfolded as follows. First, items for which the I-CVI was below the standard (items 2, 3, 15, and 16 in the initial version) were revised to items 2, 3, 16, and 19 in the revised version. For example, item 3 now describes the conditions under which it is recommended to set the learning goals, as outlined in Classification B, in plain and clear language, and a diagram.

Second, even items (and their corresponding manual) with an I-CVI above the standard value were reviewed based on comments from experts and practitioner respondents. For example, item 11 was revised because many cancer survivors with early-stage cancer do not have bone metastasis, and the risks threatening cancer survivors include bone metastasis, falls, and worsening pain.

Third, for responses that could not be fully explained by the content of the item questions alone, notes were added to the manual, and they must be referenced while answering the questions. Specifically, we analyzed the cognitive interview data and attempted to revise the items for which practitioner respondents indicated unsureness about the answers. For example, items 1-3 in the revised version (i.e., also items 1-3 in the initial version) now specify that respondents must respond to the tool considering the situation at the time of the response, not at the time of setting the life goal. The manual now also clearly defines that if it is difficult to ask the cancer survivor directly whether one “wants to engage in” (item 4 in the revised version) or “think is achievable” (item 5 in the revised version) due to the patient experiencing anxiety, low motivation, or denial (i.e., defense mechanisms), practitioner respondents must select response option 3 (i.e., “neither agree nor disagree”). Practitioner respondents also commented that they were unsure how to answer items 6, 15, and 17 in the initial version (items 12, 16, and 20 in the revised version) when the life goals established were to be fulfilled after hospital discharge, as the healthcare professionals only contacted the cancer survivors during hospitalization and responded to the ReGAT-C. Therefore, these items were revised into phrases that healthcare professionals could answer during hospitalization. Items 12, 13, and 17 (items 10, 18, and 20 in the revised version) were clarified in the manual that practitioner respondents can select response option 5 (i.e., “strongly agree”) if the user did not have to do what was described in these items because the cancer survivor had a mild symptom or was discharged early. Many items were also revised to include the number of life goals in the phrase (e.g., “at least one life goal” or “all life goals”), since it was necessary to distinguish between items in which one or more of the three maximum life goals should apply and items in which all goals should apply.

Fourth, the response options for the items on Process (items 14-21 in the revised version) were revised to be similar to the options for items 2-13 in the revised version.

Fifth, three new items (items 6, 11, and 15 in the revised version) were added based on the experts’ comments on comprehensiveness. New items were not created based on the comments by novice and middle-level healthcare professional respondents on comprehensiveness because the previous revisions dealt with the issues highlighted in their comments (the first comment was tackled through changes in item 16 in the revised version, and the second comment through changes in item 8 in the revised version).

Sixth, the items were rearranged to allow practitioner respondents to answer items in order according to each subject in the tool. These efforts yielded the revised 21-item ReGAT-C (Table 8).

**Table 8:** Revised the Reengagement Life Goal Assessment Tool for Cancer Survivors (ReGAT-C) ^a^ (number): Item number of the initial ReGAT-C version; ^b^ Conditions of Classification B: It is recommended for learning goals to be set rather than performance goals when at least one of the following four conditions is met; (i) the cancer survivor does not know how to achieve their goals; (ii) the cancer survivor has a low ability to achieve their goals; (iii) the cancer survivor is more likely to become pessimistic if they fail to achieve their goals, viewing it as a serious failure; (iv) the cancer survivor does not know enough about what activities they can do because their abilities have not improved sufficiently, such as at the start of rehabilitation.

Subscales	Items	Response options
Classification	1. Number of Classification A include in the life goals (1)^a^	1: 1 unit, 2: 2 units, 3: 3 units, 4: 4 units, 5: 5 units
	2. You understand the conditions of Classification B^b^ when you set life goals. (2)	1: Strongly disagree, 2: Disagree a little, 3: Neither agree nor disagree, 4: Agree a little, 5: Strongly agree
	3. The type of life goals in Classification B and their conditions are consistent. (3)	
Characteristic	4. There is at least one life goal that the cancer survivor wants to engage in. (4)	
	5. There is at least one life goal that the cancer survivor thinks is achievable. (10)	
	6. There is at least one life goal that the cancer survivor can hold on to while undergoing cancer treatment. (newly)	
	7. The cancer survivor can act on all life goals without risk of worsening body functions. (11)	
	8. Key persons for the cancer survivor agree with the content of all life goals. (8)	
	9. There is at least one life goal that you think is achievable. (9)	
	10. There is at least one professional from a profession that differs from yours who agrees with the content of all life goals. (12)	
	11. All life goals were set after fully discussing them between the cancer survivor and you. (newly)	
	12. There is at least one life goal for which you can objectively assess the extent to which achievement has improved. (6)	
	13. There is at least one life goal that specifically defines when, who, and how. (7)	
Process	14. The cancer survivor enjoys the process of engaging in life goals. (5)	
	15. The cancer survivor feels more positive by engaging in life goals. (newly)	
	16. The cancer survivor feels either accomplishment or satisfaction by engaging in life goals. (15)	
	17. You can explain to healthcare professionals from a profession that differs from yours why you have set these life goals. (14)	
	18. After life goals were established, the goals or the related interventions were reassessed for necessary modifications or additions. (13)	
	19. You share important life goals with the cancer survivor through either the survivor or you by writing them down on paper, so that they don’t forget. (16)	
	20. You reflect with the cancer survivor on whether there has been progress toward the achievement of the life goals. (17)	
	21. The cancer survivor can recall what their life goals were. (18)	

Retests for novice and middle-level healthcare professionals (questionnaire)

All nine practitioner respondents replied, with one nurse commenting that the subject of item 18 of the revised ReGAT-C was unclear, and that information should be added. Therefore, this item was further revised to “After the life goals were established, you reassessed whether the goals or related interventions needed modifications or additions.” The other practitioner respondents stated that the revised ReGAT-C was acceptable.

Assessment of content validity by experts (second questionnaire)

All 11 experts replied. The results of their re-evaluation of the 21 items of the ReGAT-C showed that all I-CVI values ranged from 0.91 to 1.00, and S-CVI/ave values were 0.98 and 0.99, that is, all values were above the standard values (Table 5).

## Discussion

This study aimed to revise the ReGAT-C designed by the authors to have content validity as a tool for assessing the quality of life goals practice when healthcare professionals, including PTs, OTs, and RNs, set life goals with nonterminal stage cancer patients who are undergoing inpatient cancer treatment for discharge to home. The revised version of the 21-item ReGAT-C was verified to have content validity through this study.

Methodological strengths

The ReGAT-C, its manual, and the items were developed through two literature review procedures and discussions and consensus among the development group, which can increase researcher bias [27]. Therefore, to reduce researcher bias, we deemed it necessary to conduct questionnaires and interviews to obtain opinions from experts and practitioner respondents with a wide range of attributes, all of whom were recruited through purposive sampling. Furthermore, the initial version of the ReGAT-C was substantially revised through the outcomes of the aforementioned interviews and questionnaires conducted with experts and practitioner respondents; this led to the need for retesting the revised version [18]. Accordingly, the experts and practitioner respondents engaged in a retest procedure using the revised ReGAT-C through questionnaires; these processes yielded the revised version of the ReGAT-C, which can be useful for measuring the life goal-setting processes for cancer survivors in Japanese inpatient facilities.

Notes on the use of the ReGAT-C

During the interviews, experts and practitioner respondents commented that healthcare professionals had insufficient knowledge to estimate the feelings of cancer survivors while responding to items 4, 5, and 10 in the initial version (items 4, 5, and 14 in the revised version) of the ReGAT-C. This implies that users should use the ReGAT-C in combination with assessment tools based on the cancer survivor’s perspective, such as the Canadian Occupational Performance Measure [28], QoL, and the Collaborative Relationship Scale for clients and OTs [29]. Furthermore, ReGAT-C users should consider whether adequate collaboration has been achieved between them and the cancer survivors. In previous conceptual analyses of collaboration in the fields of nursing and occupational therapy, the dimensions of collaboration included the existence of something to share, teamwork, respect and trust, active participation, communication and interaction, and complementing each other [30,31]. This showcases how critical it is for cancer survivors and healthcare professionals to complement each other during the life goal-setting process and to share with one another the topics pertaining to life goals through interactive communication. These efforts may help overcome the healthcare professionals’ lack of capacity to estimate cancer survivors’ feelings. Furthermore, since these assertions were considered consistent with the experts’ arguments during interviews toward including a new item in the tool for assessing the degree of discussion between cancer survivors and healthcare professionals, item 11 was added to the revised ReGAT-C to address the topic.

Regarding the I-CVIs for the first version of the ReGAT-C, items 2 and 3 related to Classification B (learning and performance goals) were below the standard. These values were related to the analysis results of the FGI for experts; specifically, the FGI data indicated that simply distinguishing between learning and performance goals would not lead to improved quality for the life goal-setting practice and that it was difficult for tool practitioner respondents to understand clinical situations during which setting learning goals instead of performance goals would be ideal. Regarding the reasoning for including items 2 and 3 in the tool, they were developed from a literature review of rehabilitation practices that apply goal-setting theory [17]. Depending on the situation, setting performance goals can be counterproductive, as some individuals may be very negatively impacted by the perception of failure (especially in the actual case of failure) in attempting to achieve specific performance goals, rendering it optimal for learning goals to be set in such specific cases [32]. This recommendation of the last cited study was suggested in the context of physical activity and for individuals with unspecified diseases; still, there are studies showing that the goal disengagement ability (i.e., to the capacity of abandoning unattainable goals) has positive effects on cancer survivors’ QoL and/or emotions [4,33-35]. Therefore, items 2 and 3 were included in the ReGAT-C because the professionals setting learning goals together with cancer survivors may help avoid the adverse psychological effects that may occur in relation to failure to achieve specific goals. Furthermore, items 2, 3, and the manual including the conditions wherein users should set learning goals were revised to be presented in plain and clear language, and a diagram; upon these changes, the I-CVI of the revised version of the tool exceeded the standard value. This may be partially because the repetition of the implementation of the questionnaire may have enabled the experts and practitioner respondents to familiarize themselves with the tool and the distinction between learning and performance goals. This suggests that it would be advisable for healthcare professionals to use the ReGAT-C after receiving education on the topics pertaining to Classification B.

Uniqueness of the ReGAT-C

As the ReGAT-C’s content validity was confirmed, it was shown that PTs, OTs, and RNs can use it. Thus, the tool has a common language that is accessible to all three healthcare professions and showcases the potential for contribution to collaborative, multidisciplinary support efforts at improving the life goal-setting practice of cancer survivors.

There are some scales similar to the ReGAT-C, which include the GAS, Client-Centeredness of Goal Setting (C-COGS) scale, and the Self-rating Scale of Occupational Therapists for Terminal Cancer (SROT-TC). The C-COGS scale aims to evaluate client-centered goal planning (i.e., an aspect of client-centered practice) and does so by assessing, from the client’s perspective, whether the process of setting goals and the actual goals set for rehabilitation followed the client-centered approach [36]. The C-COGS scale is similar to the ReGAT-C because it focuses on approaches to enhance client participation and decision-making in rehabilitation goal setting, and on the importance, meaning, and relevance of the rehabilitation goals established for the client. However, it diverges from the ReGAT-C because the C-COGS was developed for survivors of acquired brain injury. Furthermore, in the GAS and the C-COGS scale, in which the assessor is a survivor, the psychological burden may be increased by the survivor's recall of or talk about the negative experience of cancer and its treatment. Meanwhile, the SROT-TC is a self-assessment scale developed to help OTs reflect on the overall treatment for terminal cancer patients [37]. Although the SROT-TC has similarities to the ReGAT-C in that both enable OTs to reflect on their practice, it was developed for patients with terminal cancer.

These descriptions underpin the novelty of the ReGAT-C, as it enables healthcare professionals to reflect on the life goals they collaboratively set with cancer survivors who are not in the terminal stages of cancer, have been recently diagnosed with cancer, or are under treatment. It has been reported that barriers arise under goal-setting practice with these cancer survivors, such as unknown life expectancy issues and difficulty in understanding the limitations of the life-sustaining treatment [38,39]. This allows for inferring that healthcare professionals need to reflect on their practices related to life goal setting while attempting to minimize the burden on cancer survivors, and the ReGAT-C stands to contribute to these efforts.

Limitations and future directions

The first limitation of this study is that the members of the ReGAT-C development group did not include PTs. This could lead to the ReGAT-C not adequately representing PTs' specific life goal-setting practices. Questionnaires and interviews were conducted with a total of seven experts and practitioner respondents who were PTs to compensate for this. Particularly, four PT experts contributed to ensuring the content validity of the ReGAT-C.

Second, the coding during the qualitative analysis was not performed by independent researchers. Still, the COSMIN [18] emphasizes the importance of involving at least two or more researchers in the analysis, even if the qualitative analysis is not necessarily conducted between independent researchers during content validity testing. We adhered to COSMIN’s recommendations by involving all three authors in discussions about the qualitative analysis results, albeit it was only the first author who performed the coding procedures.

Third, this study was not validated for use in other countries because the data were collected only from healthcare professionals in Japan. Because the ReGAT-C targets cancer survivors undergoing inpatient treatment with the expectation of hospital discharge, it is predicted that the results of this study will not be thoroughly applicable to countries other than Japan, which have different medical insurance systems and hospitalization durations for cancer.

Despite these limitations, the examinations as to the content validity of the ReGAT-C significantly advance the literature and practice by delivering a tool that can be used to secure a high-quality life goal-setting practice for both cancer survivors and healthcare professionals. Since life goal achievement and importance have been shown to correlate with QoL among cancer survivors [4], after clarifying the construct validity, criterion validity, and retest reliability of the ReGAT-C, further studies investigating the correlation between the ReGAT-C and QoL are warranted.

## Conclusions

This study conducted questionnaires and interviews with experts and novice and middle-level healthcare professionals with the aim of confirming the content validity of and revising the initial 18-item version of the ReGAT-C, a tool for assessing the quality of life goal-setting practice of nonterminal cancer survivors. The revised version of the ReGAT-C comprises 21 items and was verified to have content validity. This tool is expected to make possible the reflection of healthcare professionals on their practices regarding life goal setting, and to promote collaboration among these professionals, cancer survivors, and multidisciplinary teams, and to facilitate the minimization of the burden on cancer survivors during life goal-setting practice.
